# Multicenter Phase II study of FOLFOX or biweekly XELOX and Erbitux (cetuximab) as first-line therapy in patients with wild-type *KRAS*/*BRAF* metastatic colorectal cancer: The FLEET study

**DOI:** 10.1186/s12885-015-1685-z

**Published:** 2015-10-14

**Authors:** Hitoshi Soda, Hiromichi Maeda, Junichi Hasegawa, Takao Takahashi, Shoichi Hazama, Mutsumi Fukunaga, Emiko Kono, Masahito Kotaka, Junichi Sakamoto, Naoki Nagata, Koji Oba, Hideyuki Mishima

**Affiliations:** 1 Department of Surgery, Showa University Fujigaoka Hospital, Yokohama, Japan; 2 Cancer Treatment Center, Kochi Medical School Hospital, Kochi University, Kohasu, Oko-cho, Nankoku, Kochi 783-8505 Japan; 3 Osaka Rosai Hospital, Sakai, Japan; 4 Department of Surgical Oncology, Gifu University Hospital, Gifu, Japan; 5 Department of Digestive Surgery and Surgical Oncology, Yamaguchi University, Graduate School of Medicine, Ube, Japan; 6 Sakai City Hospital, Sakai, Japan; 7 Japan Community Health care Organization Osaka Hospital, Osaka, Japan; 8 Sano Hospital, Kobe, Japan; 9 Tokai Central Hospital, Kakamigahara, Japan; 10 Kitakyushu General Hospital, Kitakyushu, Japan; 11 Department of Biostatistics, School of Public Health, Graduate School of Medicine, The University of Tokyo, Tokyo, Japan; 12 Aichi Medical University, Nagakute, Japan

## Abstract

**Background:**

The clinical benefit of cetuximab combined with oxaliplatin-based chemotherapy remains under debate. The aim of the present multicenter open-label Phase II study was to explore the efficacy and safety of biweekly administration of cetuximab and mFOLFOX-6 or XELOX as first-line chemotherapy in patients with metastatic colorectal cancer.

**Methods:**

Sixty-two patients with previously untreated *KRAS*/*BRAF* wild-type metastatic colorectal cancer were recruited to the study between April 2010 and May 2011. Patients received one of two treatment regimens, either cetuximab plus mFOLFOX-6 (FOLFOX + Cmab) or cetuximab plus biweekly XELOX (XELOX + Cmab), according to their own preference. Treatment was continued until disease progression or the appearance of intolerable toxicities. The primary endpoint was response rate; secondary endpoints were progression-free survival, overall survival, disease control rate, dose intensity, conversion rate to surgical resection, and safety.

**Results:**

The response rates in the FOLFOX + Cmab (*n* = 37) and XELOX + Cmab (*n* = 25) groups were 64.9 % (24/37) and 72.0 % (18/25), respectively. The median PFS in the FOLFOX + Cmab and XELOX + Cmab groups was 13.1 months (95 % confidence interval [CI] 12.1–17.5) and 13.4 months (95 % CI 10.1–17.9), respectively. Neutropenia was the most frequent grade 3/4 adverse event in both groups (33.9 %), followed by anorexia, acneiform eruption, skin fissure and paronychia. A waterfall plot of tumor diameter showed prominent shrinkage of the tumors in 88.7 % of patients.

**Conclusions:**

The results of the present study indicate that biweekly cetuximab plus mFOLFOX-6/XELOX is an effective and tolerable treatment regimen. Biweekly administration of cetuximab requires only one hospital visit every 2 weeks, and may become a convenient treatment option for patients with *KRAS*/*BRAF* wild-type metastatic colorectal cancer.

**Trial registration:**

This study is registered with University Hospital Medical Information Network (UMIN 000003253). Registration date is 02/24/2010.

**Electronic supplementary material:**

The online version of this article (doi:10.1186/s12885-015-1685-z) contains supplementary material, which is available to authorized users.

## Background

Cetuximab is a monoclonal antibody that targets the extracellular domain of epidermal growth factor receptor and modulates the proliferation of tumor cells. Cetuximab, in combination with irinotecan-based chemotherapy or as monotherapy, prolongs the survival of patients with metastatic colorectal cancers (mCRC) [1, 2]. However, clinical trials examining the benefit of adding cetuximab to oxaliplatin-based regimens as first-line therapy have reported inconsistent results. For example, the Oxaliplatin and Cetuximab in First-Line Treatment of Metastatic Colorectal Cancer (OPUS) study and subsequent updates demonstrated a superior response rate (RR) and progression-free survival (PFS), as well as favorable overall survival (OS), in the cetuximab plus FOFOX-4 group compared with FOLFOX-4 alone [3, 4]. Similarly, a retrospective pooled analysis of the Cetuximab Combined with Irinotecan in First-line Therapy for Metastatic Colorectal Cancer (CRYSTAL) and OPUS randomized clinical trials showed that adding cetuximab to standard first-line chemotherapy (FOLFIRI and FOLFOX) had a significant survival benefit [5].

Conversely, the COIN trial, a large prospective study investigating the potential benefit of adding cetuximab to oxaliplatin-based therapy (FOLFOX and triweekly XELOX) in v-Ki-ras2 Kirsten rat sarcoma viral oncogene homolog (*KRAS*) wild-type mCRC, failed to confirm any additional beneficial effects [6]. However, there are several concerns with that study, including a protocol amendment to reduce the dose of capecitabine only in the XELOX plus cetuximab group, a significantly smaller dose intensity of oxaliplatin in the XELOX plus cetuximab group compared with the corresponding control group [7], and significantly fewer patients receiving second-line treatment in the combination therapy group than in the chemotherapy alone group [6]. These differences that were generated during the trial may have obscured the beneficial effects of combination therapy on PFS and OS despite the better RR. Because of the currently limited range of therapeutic treatments available for mCRC, we believe further studies are needed to clarify whether the addition of cetuximab to oxaliplatin-based chemotherapy will provide further benefits.

From another perspective, biweekly XELOX (without molecular targeting therapy) has been reported to have similar antitumor activity and safety profile to triweekly XELOX [8, 9]. Likewise, the efficacy and safety of biweekly cetuximab have been shown to be comparable with those of the weekly regimen [10, 11]. In terms of convenience for patients, a 2-week cycle of intravenous oxaliplatin well matches biweekly administration of cetuximab. Considering that curing metastatic colorectal cancer remains difficult in most patients, in addition to treatment efficacy, the safety and convenience of any treatment modality are of considerable importance. Thus, to determine the efficacy of the addition of biweekly cetuximab, we undertook a Phase II trial of biweekly cetuximab plus biweekly XELOX or mFOLFOX-6 as first-line therapy for mCRC. On the basis of results from the COIN trial, in which the dose of capecitabine was reduced in the triweekly XELOX and cetuximab group, the dose of capecitabine used in the present study was adjusted and the study was performed in a non-randomized manner.

## Methods

### Patient eligibility

Patients with *KRAS* codon 12, 13, 61 wild-type metastatic colorectal cancer who had not been treated previously with chemotherapy, with the exception of adjuvant chemotherapy with oral fluoropyrimidines, were enrolled in the present study. In addition, patients with *BRAF* (V600E) mutation metastatic colorectal cancer (mCRC) were excluded from the study because of the possible relationships between the *BRAF* (V600E) mutation of mCRC and a poor response to cetuximab treatment, and/or the mutation and a poor prognosis [12–14]. Patients were eligible for inclusion if they were ≥20 years of age, had histologically confirmed colorectal cancer, had Eastern Cooperative Oncology Group (ECOG) performance status ≤1, had at least one radiologically measurable lesion, had an estimated life expectancy of ≥3 months, and had adequate function of their vital organs, including liver, kidney and bone marrow. Patients with metastatic tumor recurrence >24 weeks after adjuvant chemotherapy with an oxaliplatin-base regimen were also eligible for inclusion in this study.

### Study design

**Fig. 1:**
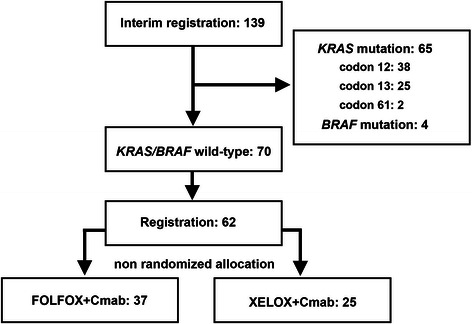
Disposition of patients. In all, 139 patients were analyzed for *KRAS* and *BRAF* mutation status. Of these, 70 patients (50.4 %) had both *KRAS* and *BRAF* wild-type metastatic colorectal cancer, and 62 fulfilled the inclusion criteria for the study. Of the 62 patients enrolled in the study, 37 chose to receive mFOLFOX-6 plus cetuximab treatment (FOLFOX + Cmab), whereas 25 chose XELOX plus cetuximab treatment (XELOX + Cmab)

This study was a single-arm Phase II open-label non-randomized multi-institutional clinical trial investigating the efficacy and safety of biweekly cetuximab plus either mFOLFOX-6 (FOLFOX + Cmab) or biweekly XELOX (XELOX + Cmab) as first-line therapy for metastatic colorectal cancer. This study is registered with the University Hospital Medical Information Network (UMIN) in Japan (study ID: UMIN 000003253, Date: 02/24/2010). The study was performed after approval by the Institutional Ethics Review Board of Yamaguchi University (ID: H22-13), as well as approval from relevant institutional review boards (IRBs) from each of the participating institutes (see Additional file 1), and was conducted in accordance with the Declaration of Helsinki. Written informed consent was obtained from each patient prior to their enrolment in the study. Patients were recruited to the study between April 2010 and May 2011, and one of two chemotherapy regimens was chosen based on individual patient preferences (Fig. 1). On Day 1 of the 2-week treatment cycle, patients in both groups received cetuximab (500 mg/m^2^ over 2 h, followed by the same dose at a rate of <10 mg/min thereafter). In the FOLFOX + Cmab group, the mFOLFOX-6 regimen consisted of oxaliplatin 85 mg/m^2^ over 2 h, folinic acid 400 mg/m^2^ over 2 h, and bolus administration of 5-fluorouracil (5-FU) 400 mg/m^2^ on Day 1, followed by continuous infusion of 2400 mg/m^2^ 5-FU over 46 h. In the XELOX + Cmab group, the biweekly XELOX regimen consisted of oxaliplatin 85 mg/m^2^ over 2 h on Day 1 and oral capecitabine 1000 mg/m^2^ twice daily on Days 1–7. Both treatment regimens were continued until disease progression or the appearance of intolerable toxicity.

Physical examination and clinical data were collected prospectively for analysis before each treatment cycle. Adverse events were monitored and evaluated according to the National Cancer Institute Common Terminology Criteria for Adverse Events (NCI-CTCAE) version 4.0. Criteria for continuation of treatment were: leukocytes >3000 /mm^3^, neutrophils >1500 /mm^3^, thrombocytes >7.5 × 10^4^ /mm^3^; alanine aminotransferase (ALT), aspartate aminotransferase (AST), serum total bilirubin and creatinine levels less than two times the upper limit of normal (ULN); vomiting, nausea and diarrhea less than grade 1; and hand–foot syndrome less than grade 2 for chemotherapy. Additional criteria for continuation of treatment included serum magnesium levels >0.7 mg/dL and cetuximab-related dermatological adverse events less than grade 2. When there was a delay in treatment of >14 days, treatment according to the study protocol was discontinued.

The primary endpoint of the present study was objective RR assessed using the Response Evaluation Criteria in Solid Tumors (RESIST) version 1.1 [15]. Tumors were evaluated by computed tomography immediately before initiation of treatment (within 2 weeks), 4 weeks after the initial treatment, and then at <8-weekly intervals thereafter. Secondary endpoints included PFS, OS, disease control rate (DCR), conversion rate to curative surgery for liver metastasis, dose intensity (DI), response rate of each metastatic site, and safety.

### Dose modification

Criteria for a dose reduction of oxaliplatin or fluoropyrimidines included grade 4 leukopenia and neutropenia, grade 3/4 thrombocytopenia, grade 3 febrile neutropenia, grade 3/4 nausea, vomiting, diarrhea, anorexia and stomatitis. In the case of Grade 2 peripheral neuropathy, the oxaliplatin dose was reduced. In the case of grade 3/4 acne-like rash and hand–foot syndrome, doses of cetuximab and capecitabine were reduced, respectively. When a grade 1 or 2 infusion reaction to oxaliplatin or cetuximab was observed, the infusion rate was reduced to <20 mg/min. For grade 3 or 4 infusion reactions, treatment according to the study protocol was discontinued. First and second dose reductions were by approximately 20 % and 40 % of the initial dose, respectively. When adverse events necessitating further dose reductions occurred, treatment according to the study protocol was discontinued.

### *KRAS* and *BRAF* mutations

DNA was extracted from formalin-fixed, paraffin-embedded tumor tissues. Mutations of *KRAS* at codons 12, 13, and 61 and *BRAF* mutations at codon 600 were detected by direct sequencing, as described previously [16, 17].

### Statistical analysis

Statistical analyses were performed using SAS version 9.2 (SAS Institute Inc., Cary, NC, USA). The target sample size of 57 (52 eligible patients and 10 % ineligible patients) was based on expected and threshold response rates of 62 and 40 %, respectively, with α = 0.05 and β = 0.1. *P <* 0.05 was considered significant. Graphs were created using KaleidaGraph version 4.0 (Synergy Software, Reading, PA, USA), Photoshop CS2 (Adobe Systems, San Jose, CA, USA) and IBM SPSS Statistics 19 (IBM, Armonk, NY, USA).

## Results

### Patient recruitment and characteristics

Between April 2010 and May 2011, 139 patients from 34 institutions were provisionally registered as candidates for this study (Fig. 1). Analysis of *KRAS* and *BRAF* mutations identified 69 cases of *KRAS* or *BRAF* mutations, most of which were observed at codons 12 and 13 of *KRAS* (38 and 25 cases, respectively). Mutations of codon 61 were identified in two patients only, whereas four patients were found to have *BRAF* (V600E) mutations.

Seventy patients (50.4 %) had both *KRAS* and *BRAF* wild-type metastatic colorectal cancer, and 62 patients fulfilled the inclusion criteria (45.2 % [28/62] female; median age 66 years [range 34–83 years]; Table 1) and were enrolled in the present study. Of these, 37 patients were treated with the mFOLFOX-6 plus cetuximab regimen (FOLFOX + Cmab group), whereas 25 patients chose XELOX plus cetuximab treatment (XELOX + Cmab group). The liver was the most common site of metastasis (*n* = 47; 75.8 %) and one patient had metastases to more than one site.

**Table 1:** Characteristics of patients with *KRAS* and *BRAF* wild-type metastatic colorectal cancer treated with either mFOLFOX-6 plus cetuximab (FOLFOX + Cmab) or XELOX plus cetuximab (XELOX + Cmab)

	All patients (*n* = 62)	FOLFOX + Cmab (*n* = 37)	XELOX + Cmab (*n* = 25)	*P*-value
No. women	28 (45.2 %)	18 (48.9 %)	10 (40.0 %)	0.502
Median (range) age (years)	66 (34–83)	67 (45–83)	66 (34–83)	0.769
ECOG performance status				0.885
0	55 (88.7 %)	33 (89.2 %)	22 (88.0 %)	
1	7 (11.3 %)	4 (10.8 %)	3 (12.0 %)	
Primary tumor site				0.193
Colon	36 (58.1 %)	18 (48.6 %)	8 (32.0 %)	
Rectum	26 (41.9 %)	19 (51.4 %)	17 (68.0 %)	
Metastatic site				
Liver	47 (75.8 %)	29 (78.4 %)	18 (72.0 %)	0.136
Lymph node	18 (29.0 %)	13 (35.1 %)	5 (20.0 %)	
Lung	15 (24.2 %)	10 (27.0 %)	5 (20.0 %)	
Other	8 (12.9 %)	2 (5.4 %)	6 (24.0 %)	

Unless indicated otherwise, data show the number of patients in each group, with percentage in parentheses

*ECOG* Eastern Cooperative Oncology Group

### Treatment efficacy

**Fig. 2:**
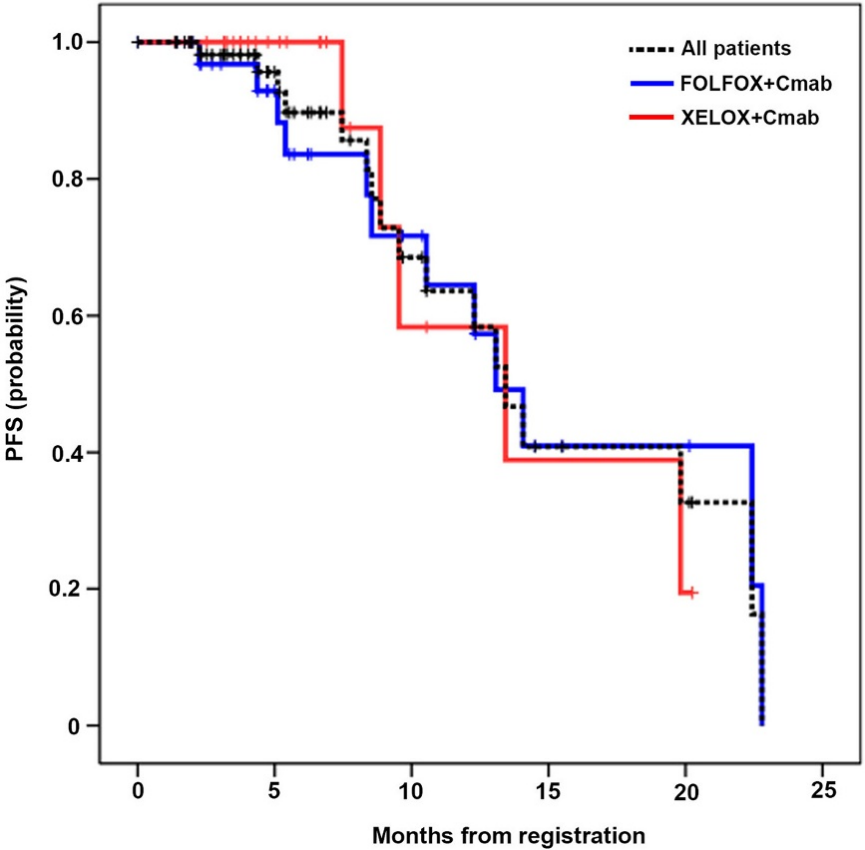
Progression-free survival (PFS). PFS is shown for patients treated with mFOLFOX-6 plus cetuximab (FOLFOX + Cmab) or XELOX plus cetuximab (XELOX + Cmab). A log-rank test did not reveal any significant differences in PFS between the two groups (*P* = 0.99)

**Fig. 3:**
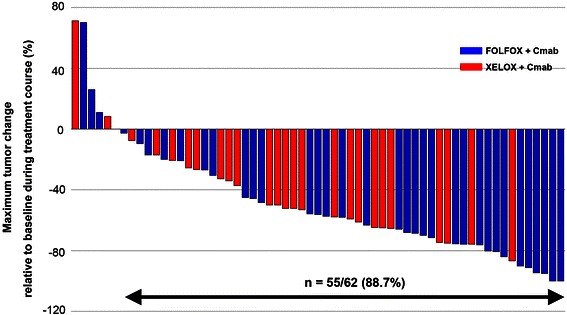
Maximum change in tumor size from baseline in individual patients over the course of treatment. Changes in tumor size (diameter) were assessed in patients treated with mFOLFOX-6 plus cetuximab (FOLFOX + Cmab) or XELOX plus cetuximab (XELOX + Cmab). Tumor shrinkage relative to baseline was observed in 88.7 % of patients, revealing the strength of combination therapy with cetuximab and oxaliplatin-based chemotherapy

Overall, two complete and 40 partial responses were observed (Table 2), and the RR for both groups was 67.7 %. Because 15 patients (24.2 %) had stable disease, the DCR was 91.9 %. There was no significant difference in the response rate between the FOLFOX + Cmab and XELOX + Cmab groups (64.9 % vs 72.0 %, respectively; *P* = 0.56, Chi-squared test). The median PFS in the FOLFOX + Cmab and XELOX + Cmab groups was 13.1 months (95 % confidence interval [CI] 11.4–18.3) and 13.4 months (95 % CI 10.1–17.9), respectively (Fig. 2). Furthermore, 88.7 % of patients exhibited prominent tumor shrinkage during treatment (Fig. 3). When response rate was assessed in patients with only one metastatic site, the response rates for liver, lung and lymph node metastases were 71.4 % (20/28), 75 % (3/4), and 57.1 % (4/7), respectively. At the final assessment, after a median follow-up of 36.3 months (95 % CI 28.4–35.6), 54.8 % of patients (34/62) had died: 88 % of the deaths were due to disease progression, and 6.5 % of patients (4/62) died of other causes, including pneumonia and unexpected accidents. The median OS in the FOLFOX + Cmab and XELOX + Cmab groups was 38.1 months (95 % CI 33.5–42.6) and 47.0 months (95 % CI 32.1–61.9), respectively.

**Table 2:** Efficacy of treatment with either mFOLFOX-6 plus cetuximab (FOLFOX + Cmab) or XELOX plus cetuximab (XELOX + Cmab)

	All patients (*n* = 62)	FOLFOX + Cmab (*n* = 37)	XELOX + Cmab (*n* = 25)
Tumor response			
CR	2	2	0
PR	40	22	18
SD	15	9	6
PD	3	2	1
NE	2	2	0
Response rate (%)	67.7 (54.7–79.1)	64.9 (47.5–79.8)	72.0 (50.6–87.9)
DCR (%)	91.9 (82.3–97.3)	89.2 (74.6–97.0)	96.0 (79.7–99.9)
Median PFS (months)	13.4 (12.1–17.5)	13.1 (11.4–18.3)	13.4 (10.1–17.9)
Median OS (months)	38.1 (30.3–45.8)	38.1 (33.5–42.6)	47.0 (32.1–61.9)

Data are given as the number of patients in each group or as median values with 95 % confidence intervals in parentheses

*CR* complete response, *PR* partial response, *SD* stable disease, *PD* progressive disease, *NE* not evaluated, *DCR* disease control rate, *PFS* progression free survival; OS, overall survival

### Exposure to treatment

The median number of treatment cycles was 10 (Table 3). The median cumulative dose of cetuximab was 6480 mg/m^2^ and the DI was 216 mg/m^2^ per week. Fifty-two of 62 patients received a relative DI >75 %. In the case of oxaliplatin, 37 of 62 patients received a relative DI >75 %. There was no significant difference in the DI of oxaliplatin between the FOLFOX + Cmab and XELOX + Cmab groups (*P* = 0.25, *t*-test).

Seventeen patients (27.4 %) discontinued treatment because of disease progression, whereas 19 patients (30.6 %) discontinued the study treatment protocol to undergo surgical resection. In nine patients (14.5 %), there was an unacceptable delay in treatment (>14 days) after a median treatment period of 5 months. One patient in the FOLFOX + Cmab group developed interstitial pneumonia, although it is not clear whether this was related to the treatment regimen.

**Table 3:** Exposure to treatment for patients treated with either mFOLFOX-6 plus cetuximab (FOLFOX + Cmab) or XELOX plus cetuximab (XELOX + Cmab)

	All (*n* = 62)	FOLFOX + Cmab (*n* = 37)	XELOX + Cmab (*n* = 25)
No. treatment cycles			
Median	10 (5.5–17.5)	10 (5–19)	9.5 (6–14)
Cetuximab treatment			
Cumulative dose (mg/m^2^)	6480 (2187–12 480)	6750 (3580–14 634)	6397 (4302–11 198)
DI (mg/m^2^ per week)	216 (189–247)	223 (189–233)	237 (179–261)
No. with relative DI >75 %	52	28	24
Oxaliplatin			
Cumulative dose (mg/m^2^)	992 (600–1812)	987 (505–2021)	1008 (661–1597)
DI (mg/m^2^ per week)	31.4 (24–39)	30 (28–35)	39 (29–42)
No. with relative DI >75 %	37	20	17
Bolus 5-FU			
Cumulative dose (mg/m^2^)		4392 (1795–9928)	
DI (mg/m^2^ per week)		135 (120–175)	
No. with relative DI >75 %		21	
5-FU Infusion			
Cumulative dose (mg/m^2^)		28414 (14750–60644)	
DI (mg/m^2^ per week)		890 (803–1062)	
No. with relative DI >75 %		21	
Capecitabine			
Cumulative dose (mg/m^2^)			23700 (14250–38925)
DI (mg/m^2^ per week)			878 (666–978)
No. with relative DI >75 %			16

Numbers in parenthesis show the interquartile range

*DI* dose intensity, *5-FU* 5-fluorouraci

### Safety and adverse events

**Fig. 4:**
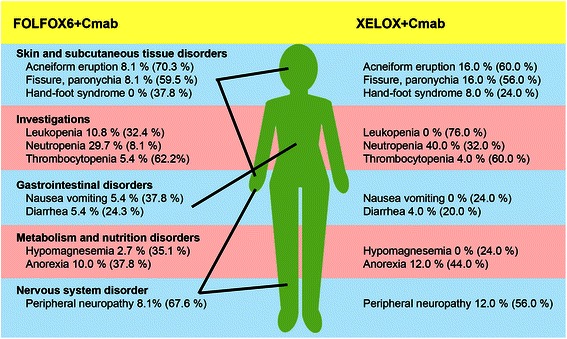
Common adverse events. Common adverse events in patients treated with either mFOLFOX-6 plus cetuximab (FOLFOX + Cmab) or XELOX plus cetuximab (XELOX + Cmab) are shown. The percentages of grade III or IV adverse events for both treatments are shown. The figures in parenthesis are the percentages of grade I or II adverse events

Common adverse events are shown in Fig. 4 (see Additional file 2 for more information). Across the entire patient cohort, the most common grade 3 and grade 4 adverse event was neutropenia, followed by acneiform eruption and paronychia. The skin eruptions related to cetuximab administration occurred at a similar rate in both groups. Grade 4 neutropenia was observed in both the FOLFOX + Cmab and XELOX + Cmab groups, whereas hand–foot syndrome was seen only in the XELOX + Cmab group. No patient died within 28 days of the last treatment cycle.

## Discussion

The present Phase II study has demonstrated the treatment efficacy and safety of biweekly cetuximab plus oxaliplatin-based chemotherapy in patients with both *KRAS* and *BRAF* wild-type mCRCr. The RR to treatment in the FOLFOX + Cmab and XELOX + Cmab groups was 64.9 % and 72.0 %, respectively. A waterfall plot showed substantial tumor shrinkage in most patients in both groups. The treatment was well tolerated, allowing a median treatment duration of 5.4 months, which corresponds to a median number of 10 treatment cycles.

Several clinical trials have demonstrated that the overall RR (or RR) to FOLFOX plus weekly cetuximab treatment varies between 57 and 72 % [3, 18–20] . Thus, the RR obtained in the present study is within the range reported previously. Furthermore, biweekly administration of cetuximab and FOLFOX was recently reported to have a similar RR [21, 22], which suggests that biweekly administration of cetuximab in combination with FOLFOX is an acceptable treatment option, and that the biological response of the tumor to this treatment regimen is similar in different studies, including the present clinical study.

In the present study, the RR to biweekly cetuximab and XELOX was 70.2 %. To our knowledge, the clinical efficacy of XELOX and cetuximab has only been reported in a few studies up until now. For example, a Swiss study reported a RR of 45.6 % to XELOX and weekly cetuximab treatment, administered regardless of the *KRAS* mutation status [23]. In the COIN trial, the overall RR to XELOX combination therapy was 51 % in patients with *KRAS* wild-type mCRC [18]. These results may have been interpreted to mean that the addition of cetuximab to XELOX is less advantageous than FOLFOX treatment. However, in the present study, an equivalent or even higher RR was obtained in the XELOX + Cmab group compared with the FOLFOX + Cmab group. Even though direct comparisons of trial results can be challenging, we consider that the findings of the present study are important because they are derived from proper dosage adjustment of capecitabine and a biweekly cycle of infusion treatment that was accompanied by pre-treatment physical and laboratory assessments.

The dose of capecitabine used in the present study was determined on the basis of results from a previous trial. In the COIN trial, patients were initially administered 1000 mg/m^2^ capecitabine twice daily for 2 weeks as part of a triweekly XELOX regimen [6]. After 73 % of all patients had been randomized, the dose of capecitabine was reduced to 850 mg/m^2^ twice daily only in the cetuximab plus XELOX treatment arm due to a higher rate of adverse events than expected. Before this protocol amendment, patients in the XELOX plus cetuximab treatment group received significantly lower doses of both oxaliplatin and capecitabine compared with the corresponding control group [6, 7], which could have obscured the benefit of combination therapy in the COIN trial. Thus, in the present study, the dose of capecitabine was set at 1000 mg/m^2^ twice daily for 1 week as part of the biweekly XELOX regimen, which is comparable to the reduced DI in the COIN trial (1000 vs. 1133 mg/m^2^ per day). Consequently, the safety profile in the XELOX + Cmab group was similar to that in the FOLFOX + Cmab group, most likely contributing to the continuation of treatment according to the study protocol and the achievement of higher RR in this study. In addition, these positive effects could explain the longer PFS in the present study compared with that reported in the COIN trial (13.4 vs. 7.4 months, respectively). The frequent use of second-line treatment in the present study, including chemotherapy (43.5 %), radiation therapy (6.5 %), and surgical resection (30.6 %), should also be taken into account. In the COIN trial, only 56 % of patients received second-line treatment, probably due to frequent adverse events. These results may have some effect on survival, and again highlight the importance of proper dose adjustment.

The median OS in the present study was 38.1 months (95 % CI 30.1–45.8). Based on the fact that 30.6 % of patients underwent surgical resection a median of 3.75 months (95 % CI 2.96–4.14) after entering the study, the patients in this study can be considered to be in a relatively early phase of advanced disease. In view of a recent report that median OS after hepatic resection for metastatic colorectal cancer exceeds 45 months [24], the results of the present study are within the expected range. Indeed, the median OS for patients who underwent surgical resection was 47.7 months (95 % CI 40.3–55.1). Moreover, the results of the present study are encouraging because the treatment regimens were found to be safe pre-operative chemotherapy regimens for liver metastasis. However, further studies in a larger number of patients are needed to confirm this.

The occurrence of hand–foot syndrome, a characteristic adverse event associated with the use of capecitabine, was well controlled in this study. In the present study, only two patients (of 25; 8.0 %) developed grade 3 hand–foot syndrome, which is lower than reported in the COIN trial (13 %), but equivalent to that reported for XELOX treatment alone [25]. Because several factors (e.g. DI, patient characteristics etc.) often differ among clinical trials, it is difficult to provide a definitive explanation for the apparent differences in results. However, the biweekly regimen generally allows for more frequent physical assessment and the provision of prophylactic information and/or treatment of any dermatological side effects than triweekly regimen. Consequently, the patients in the present study could have received better skin care, leading to less frequent adverse dermatological events. One concern is that grade 3/4 neutropenia occurred in as many as 40 % of patients in the XELOX + Cmab group, compared with a reported percentage of grade 3/4 neutropenia in triweekly and/or biweekly XELOX regimens between 3 and 10 % [6, 8, 9, 23, 25]. However, some studies in Japanese patients with colorectal cancer have also reported a high rate of grade 3/4 neutropenia with XELOX-based regimens (16–20 %) [26, 27]. Thus, differences in genetic background could explain, in part, the different adverse event profiles in Japanese patients.

One important limitation of the present study is the lack of information regarding *NRAS* mutation status. Recent retrospective studies analyzing samples from prospective clinical trials, most of which were published after the initiation of the present Phase II study, have reported that mutations at *NRAS* codons 12, 13 and 61 have a negative effect on anti-EGFR antibody therapy in mCRC patients [28, 29]. Although the rate of *NRAS* mutations in mCRC in the Japanese population is not well known, based on evidence from European countries we would expect the rate to be approximately 5–10 %. The exclusion of these patients from the present study could have demonstrated a more evident effect of the treatment regimen and robust results. Thus, further studies taking *NRAS* mutation status into consideration are necessary.

## Conclusions

A Phase II study was conducted to explore the safety and efficacy of biweekly administration of cetuximab combined with mFOLFOX-6 or biweekly XELOX in patients with *KRAS* codon 12, 13, 61 wild-type and *BRAF* (V600E) wild-type mCRC. Despite the small number of patients recruited to the study and the strict selection criteria, the results suggest that the treatment regimens evaluated herein exhibit similar efficacy to established treatment regimens, and have an acceptable safety profile. In particular, the findings of the present study are important in that we demonstrate that the combination of a biweekly cycle of XELOX plus cetuximab resulted in quite a high RR because of appropriate dose adjustment and more frequent evaluation for and treatment of adverse skin effects. Further studies comparing the treatment efficacy of mFOLFOX-6 plus cetuximab with that of a biweekly XELOX plus cetuximab regimen, focusing on OS and ideally randomized in nature, are warranted in order to establish optimal treatment strategies.

## Additional files

Additional file 1: **Table listing the Central ethics committee and the IRBs of the participating institutes.** The IRB of each participating institute reviewed and approved the protocol of the present study. (DOCX 17 kb)
Additional file 2: **Adverse events in patients treated with cetuximab and oxaliplatin-based chemotherapy.** (DOCX 16 kb)
