# Supplementary material for: Multicenter Phase II study of FOLFOX or biweekly XELOX and Erbitux (cetuximab) as first-line therapy in patients with wild-type KRAS/BRAF metastatic colorectal cancer: The FLEET study
Source: BMC Cancer. 2015 Oct 14;15:695. doi: 10.1186/s12885-015-1685-z (PMC4607014; doi:10.1186/s12885-015-1685-z)
Supplement: Additional file 1: — Table listing the Central ethics committee and the IRBs of the participating institutes. The IRB of each participating institute reviewed and approved the protocol of the present study. (DOCX 17 kb) [file 12885_2015_1685_MOESM1_ESM.docx]

**Additional File 1**

**Central ethics committee and the participating institutes.**

| **Institutions** | **Address** | **Zip code** |
| --- | --- | --- |
| ***Central ethics committee*** | | |
| Yamaguchi University Hospital | 1-1-1 Minamikogushi, Ube, Yamaguchi | 755-0046 |
| ***Participating institutes*** | | |
| National Hospital Organization Osaka National Hospital | 2-1-14 Hoenzaka, Chuo-ku , Osaka-shi, Osaka | 540-0006 |
| Kitakyushu General Hospital | 5-10-10 Yugawa, Kokuraminami-ku, Kitakyushu-shi, Fukuoka | 800-0295 |
| Yokoyama Hospital for Gastroenterological Diseases | 3-11-20 Chiyoda, Naka-ku, Nagoya-shi, Aichi | 460-0012 |
| Matsunami General Hospital | 185-1 Dendai, Kasamatsu, Hashima, Gifu | 501-6062 |
| Sano Hospital | 2-5-1 Shimizugaoka, Tarumi-ku Kobe-shi, Hyogo | 655-0031 |
| Kobe Ekisaikai Hospital | 1-21-1 Manabigaoka, Tarumi-ku, Kobe-shi, Hyogo | 655-0004 |
| Osaka City University Graduate School and Faculty of Medecine | 1-5-17 Asahimachi, Abeno-ku, Osaka-shi, Osaka | 545-8586 |
| Nakadori Generall Hospital | 3-15 Minamidorimisonomachi, Akita-shi, Akita | 010-8577 |
| JCHO Osaka Hospital | 4-2-78 Fukushima, Fukushima-ku, Osaka-shi, Osaka | 591-8025 |
| Shiga Medical Center for Adults | 5-4-30 Moriyama, Moriiyama-shi, Shiga | 524-8524 |
| Kawasaki Saiwai Hospital | 31-27 Oomiyacho, Saiwaiku, Kawasaki, Kanagawa | 212-0021 |
| Mito Medical Center | 280 Sakuranosato, Ibaraki-cho, Higashiibaraki-gun, Ibaraki | 311-3193 |
| Toyonaka Municipal Hospital | 4-14-1 Shibahara-cho, Toyonaka-shi, Osaka | 560-8565 |
| Showa University Fujigaoka Hospital | 1-30 Gujigaoka, Aoba-ku, Yokohama-shi, Kanagawa | 227-8501 |
| National Hospital Organization Kobe Medical Center | 3-1-1 Nishiochiai, Suma-ku, Kobe-shi, Hyogo | 654-0155 |
| Kochi Medical School Hospital | 185-1 Kohasu,Oko-cho,Nankoku-shi,Kochi | 783-0043 |
| Kagawa University Hospital | 1750-1 Ikenobe, Miki-cho, Kita-gun, Kagawa | 761-0701 |
| Osaka Rosai Hospital | 1179-3 Nagasone-cho, Kita-ku, Sakai-shi, Osaka | 591-8025 |
| Kanazawa Medical University Hospital | 1-1 Daigaku, Uchinada-cho, Kahoku-gun, Ishikawa | 920-0293 |
| Japanese Red Cross Kanazawa Hospital | 2-251 Minma, Kanazawa-shi, Ishikawa | 921-8162 |
| Saiseikai Shiga Hospital | 2-4-1 Ohashi, Ritto-shi, Shiga | 520-3046 |
| Hokkaido Cancer Center | 2 Chome-3-54 Kikusui 4 Jo, Shiroishi-ku, Sapporo-shi, Hokkaido | 003-0804 |
| Fukui-Ken Saiseikai Hospital | 7-1 Funabashi, Wadanaka-cho, Fuku-shi, Fukui | 918-8503 |
| Kanazawa University Hospital | 13-1 Takara-machi Kanazawa-shi, Ishikawa | 920-8641 |
| Sasitama Medical University | 1981 Kamodatsujidomachi, Kawagoe-shi, Saitama | 350-8550 |
| Japanese Red Cross Kyoto Daiichi Hospital | 15-749 Honmachi, Higasiyama-ku, Kyoto-shi, Kyoto | 605-0981 |
| Toyama Prefectural Central Hospital | 2-2-78 Nishinagae, Toyama-shi, Toyama | 930-0975 |
| Fukui Prefectural Hospital | 2-8-1 Yotsui, Fukui-shi, Fukui | 910-8526 |
| Ishikawa Prefectural Central Hospital | 2-1 Kuratsukihigashi, Kanazawa-shi, Ishikawa | 920-8530 |
| Sakai City Medical Center | 1-1-1 Ebarajicho, Nishi-ku, Sakai-shi, Osaka | 593-8304 |
| Hyogo Prefectural AWAJI Medical Center | 1-1-137 Shioya, Sumoto-shi, Hyogo | 656-0021 |
| Minoh Cith Hospital | 5-7-1 Kayano, Minoo-shi, Osaka | 562-0014 |
| Gifu University Hospital | 1-1 Yanagido, Gifu-shi, Gifu | 501-1112 |
| Ikeda City Hospital | 3-1-18 Jonan, Ikeda-shi, Osaka | 563-8510 |
| Kitano Hospital | 2-4-20 Ogimachi, Kita-ku, Osaka-shi, Osaka | 530-8480 |
| Osakakita Teishin Hospital | 1-1-6 Nakazaki, Kita-ku, Osaka-shi, Osaka | 530-8798 |
| Ibaraki Prefectural Central Hospita | 6528 Koibuchi, Kasama-shi, Ibaraki | 309-1600 |
| University of Occupational and Environmental Health, Japan | 1-1 Iseigaoka, Yahata-nishi-ku, Kitakyushu, Fukuoka | 807-8555 |
| Teikyo University Chiba Medical Center | 3426-3 Anesaki, Ichihara-shi, Chiba | 299-0111 |
| Otsu Municipal Hospital | 2-9-9 Motomiya, Otsu-shi, Shiga | 520-0804 |
| Yodogawa Christian Hospital | 1-7-50, Kunijima, HigashiYodogawa-ku, Osaka | 533-0024 |
| Kanagawa Cancer Center | 2-3-2 Nakao, Asahi Ward, Yokohama, Kanagawa | 241-0815 |
| Gifu Municipal Hospital | 7-1 Kashimacho, Gifu-shi, Gifu | 500-8513 |
| National Hospital Organization Nagoya Medical Center | 4-1-1 Sannomaru, Naka-ku, Nagoya-shi, Aichi | 460-0001 |
| Japanese Red Cross Fukui Hospital | 2-4-1 Tsukimi, Fukui-shi, Fukui | 918-8501 |
| Toho University Sakura Medhical Center | 564-1 Shimoshizu, Sakura-shi, Chiba | 285-8741 |
