# Supplementary material for: Multicenter Phase II study of FOLFOX or biweekly XELOX and Erbitux (cetuximab) as first-line therapy in patients with wild-type KRAS/BRAF metastatic colorectal cancer: The FLEET study
Source: BMC Cancer. 2015 Oct 14;15:695. doi: 10.1186/s12885-015-1685-z (PMC4607014; doi:10.1186/s12885-015-1685-z)
Supplement: Additional file 2: — Adverse events in patients treated with cetuximab and oxaliplatin-based chemotherapy. (DOCX 16 kb) [file 12885_2015_1685_MOESM2_ESM.docx]

**Additional File 2:** Adverse events in patients treated with cetuximab and oxaliplatin-based chemotherapy.

|  | All, (n=62) | | FOLFOX6+Cmab, (n=37) | | XELOX+Cmab, (n=25) | |
| --- | --- | --- | --- | --- | --- | --- |
|  | No. Grade 1/2/3/4 adverse events | % Grade 3 and 4 adverse events | No. Grade 1/2/3/4 adverse events | % Grade 3 and 4 adverse events | No. Grade 1/2/3/4 adverse events | % Grade 3 and 4 adverse events |
| Leukopenia | 11/20/4/0 | 6.5 | 5/7/4/0 | 10.8 | 6/13/0/0 | 0 |
| Neutropenia | 1/10/16/5 | 33.9 | 1/2/9/2 | 29.7 | 0/8/7/3 | 40.0 |
| Thrombocytopenia | 30/8/3/0 | 4.8 | 17/6/2/0 | 5.4 | 13/2/1/0 | 4.0 |
| Nausea, vomiting | 15/5/2/0 | 3.2 | 10/4/2/0 | 5.4 | 5/1/0/0 | 0 |
| Anorexia | 15/10/7/0 | 11.3 | 9/5/4/0 | 10.8 | 6/5/3/0 | 12.0 |
| Diarrhea | 9/6/3/0 | 4.8 | 7/3/2/0 | 5.4 | 2/3/1/0 | 4.0 |
| Acneiform eruption | 23/18/7/0 | 11.3 | 15/11/3/0 | 8.1 | 8/7/4/0 | 16.0 |
| Fissure, paronychia | 12/24/7/0 | 11.3 | 8/14/3/0 | 8.1 | 4/10/4/0 | 16.0 |
| Peripheral neuropathy | 27/12/6/0 | 9.7 | 14/11/3/0 | 8.1 | 13/1/3/0 | 12.0 |
| Hand–foot syndrome | 11/9/2/0 | 3.2 | 11/3/0/0 | 0 | 0/6/2/0 | 8.0 |
| Hypomagnesemia | 17/2/0/1 | 1.6 | 11/2/0/1 | 2.7 | 6/0/0/0 | 0 |
